# Clinical guidelines for the prevention and treatment of osteoporosis from the Italian Society for Orthopaedics and Traumatology: preface

**DOI:** 10.1007/s10195-017-0475-6

**Published:** 2017-10-20

**Authors:** Giuseppe Sessa

**Affiliations:** Department of Orthopaedics, University Hospital Vittorio Emanuele, Catania, Italy

A population aging process is over in Italy, as in most developed countries. Over time, the average age has risen and the percentage of the population above retirement age has increased. This change is bound to have significant consequences for society, families and individuals. The increase in life expectancy will increase the cost of care, as people aged >65 years are using more health services than those aged <65 years. The higher incidence of age-related diseases, such as cancer, cardiovascular disease and dementia, involves the high cost associated with senile illnesses. It is estimated that in 2050, over 33% of the general population will be aged >65 years, compared to the current 21%. Life expectancy in 2050 will be 85.3 years for men and 90.2 years for women. This involves an increase of the frail elderly, i.e., older people who are chronically affected by multiple pathologies, with an unstable, often disabled, state of health. Frailty has a high risk of rapid deterioration of health and functional status, including diseases such as sarcopenia and osteoporosis. Aging of the Italian population, with an increase of life expectancy by 20–26% in the last 20 years [1], has led to a higher incidence and prevalence of age-related diseases, such as osteoporosis. In Italy, osteoporosis potentially affects 5,000,000 people, of whom 80% are women of post-menopausal age. In particular, it is estimated that 1 out of 3 women and 1 out of 8 men in the >50 population are affected by this disease. Osteoporosis is characterized by an increased risk of experiencing a ‘fragility fracture’. Osteoporosis fractures can lead to important consequences, such as hospitalization followed by long periods of immobility, need of surgical treatment, increased disability and partial or complete loss of autonomy in daily activities [2]. It must then be considered that the presence of a fragility fracture represents a major risk factor for a subsequent fracture, with rates increasing by 2- to 5-fold, as reported by an Italian group [3]. The demographic structure of a country and the analysis of its perspective evolution, are the basis for a correct health policy [4]. Osteoporosis is a priority that the Italian National Health System has to face urgently. An active, not ‘on demand,’ medical service requires intense prevention and long-term care campaigns. Efficient and accurate diagnostic and therapeutic programs, based on comprehensive knowledge of bone tissue genetics, pathophysiology and metabolism, constitute a fundamental support to clinical practice. Fragility fractures represent a dramatic epilogue in the natural history of osteoporosis, as they undermine a patient’s quality of life while burdening the health system. Costs are very high; estimates for the year 2002, in fragility fracture patients aged >65 years, show that expenses are related both to surgical treatment and rehabilitation, while total costs for the year 2006 are estimated to be about 1,000,000,000 € in the same population. Despite its high prevalence (4.7 million people affected in Italy), osteoporosis is perceived as a less severe disease, with respect to acute myocardial infarction and diabetes, both by public opinion and primary care physicians [5]. Consequently, often incorrect and superficial management has led to an increase in fracture incidence and, as a domino effect, in the incidence of re-fractures. Today, fragility fractures still represent an underdiagnosed and undertreated condition [6]. The orthopaedic surgeon is often the first doctor who should recognize bone fragility. This is the basis for the Italian Society of Orthopaedics and Traumatology (SIOT; Società Italiana di Ortopedia e Traumatologia) to develop guidelines on osteoporosis and bone fragility for its members [7].

The document predisposed by a multidisciplinary group of experts represents a useful component in the efforts made in Italy in the past decade. The recommendations were constructed on levels of evidence, based on published works, with tool-boxes useful for a quick consultation. Moreover, the proposal for the creation of Fracture Units (or Fracture Liaison Services) for the therapeutic continuity of bone fragile patients is generously presented and discussed, in an unprecedented way. We are confident that our efforts will be appreciated by our readers.
